# German veterinarians asked: a cross-sectional study on microbiological examination and antimicrobial use in canine reproductive medicine

**DOI:** 10.3389/fvets.2025.1645496

**Published:** 2025-07-29

**Authors:** Alicia Rojahn, Anna Sophia Leps, Sandra Goericke-Pesch

**Affiliations:** Unit for Reproductive Medicine – Clinic for Small Animals, University of Veterinary Medicine Hannover, Foundation, Hannover, Germany

**Keywords:** canine reproduction, vaginal bacterial culture, antimicrobial use, antimicrobial resistance, breeding management

## Abstract

Despite the growing global threat of antimicrobial resistance, many dog breeders still request antimicrobial treatment prior to mating, often based on vaginal bacterial culture examinations. However, several previous studies failed to identify differences in the vaginal microbiota between healthy dogs and those with reproductive tract disorders; thus, treating healthy bitches with antimicrobials regardless of the bacterial findings is contraindicated. To investigate current practices of German small animal veterinarians regarding microbiological sampling and antimicrobial treatment in canine reproductive medicine, we conducted an online survey using LimeSurvey®. The questionnaire included questions (single/multiple choice) about procedures of microbiological swab sampling and handling in general and in canine reproductive medicine specifically, as well as on antimicrobial use in the respective field. The objective was to identify educational and research needs regarding vaginal bacterial culture interpretation and antimicrobial treatment in canine breeding. We found that treating clinically healthy breeding bitches remains common practice among both breeders and veterinarians. Our findings highlight the importance of continuous education and improved communication to reduce inappropriate antimicrobial use in canine reproduction. Furthermore, research on the reproductive microbiome in relation to fertility is essential for evidence-based treatment decisions.

## 1 Introduction

Vaginal bacterial culture examinations and antimicrobial treatments prior to mating are frequently performed as part of breeding management in canine reproductive medicine. Treatment is often based on the identification of bacteria by culture-based techniques in general. However, it is well known that the female genital tract is not sterile (1, 2), and neither is the canine one (3–9). Certain opportunistic bacteria, e.g., *Escherichia* (*E*.) *coli* and *Streptococcus* (*Sc*.) *canis* (10–12), are commonly identified within the vaginal flora of healthy bitches (3–8). Moreover, several previous studies failed to identify differences in the composition of the vaginal microbiota between healthy dogs and those with clinical abnormalities such as vaginitis or infertility (12–17). In addition, even bacterial monocultures with high-grade growth could be considered physiological in healthy animals and do not necessarily indicate a (subclinical) infection (3, 7, 8). This challenges the appropriate interpretation of bacterial culture findings and leads to uncertainty among veterinarians when determining the need for antimicrobial treatment. Furthermore, recent studies using 16S-rDNA-sequencing indicate that conventional culture-based methods vastly underestimate microbial diversity, detecting only up to 10% of the microbiota (18–20). Therefore, the relevance of testing clinically healthy dogs is being questioned. Nevertheless, many breeders still request vaginal swab sampling and antimicrobial prescriptions due to established habits and concerns about “infectious infertility.”

In practice, antimicrobials are often administered “just in case” as “treatment cannot hurt.” Veterinarians may be influenced by breeders or might even feel pressured to prescribe antibiotics (21), as they do not want to be held responsible for unsuccessful breeding. This is particularly challenging for veterinarians who are not specialized in reproductive medicine, as the canine reproductive microbiome remains a niche subject that has seen considerable development in recent years. However, inappropriate use of antimicrobials exacerbates antimicrobial resistance (AMR) (22–25), a global health threat associated with an estimated 1.27 million deaths worldwide yearly (23). According to the One Health approach, overuse of antimicrobials not only affects the treatment of bacterial infections in the individual animal but also impacts human health and the environment (26, 27). Furthermore, the close contact between humans and companion animals increases the risk of AMR transfer to humans, as pets act as reservoirs for resistance genes (28, 29).

Despite growing awareness of antimicrobial use, unreasonable prescriptions still occur, which contradicts antimicrobial stewardship. In a 2008 survey, 27.9% of breeders reported administering antibiotics before mating. Additionally, 55.1% stated that they routinely perform a bacteriological examination prior to breeding, while 40% of veterinarians responded that they recommended such an examination beforehand (30). Moreover, canine breeding was shown to be particularly relevant to resistance development. Intensive use of antimicrobials in breeding kennels leads to the selection and transmission of multi-resistant bacteria (31–33). Nevertheless, education and restrictive legislation have changed the handling of antimicrobials. The World Health Organization (WHO) has classified antimicrobials used in veterinary medicine in relation to their importance in human medicine as important (IA), highly important (HIA), critically important (CIA), or highest priority critically important antimicrobials (HPCIA) (34). In Germany, the amendment to the Veterinary Home Pharmacy Ordinance (Tierärztliche Hausapothekenverordnung, TÄHAV) came into force in 2018 to restrict the use of HPCIA in animals, specifically fluoroquinolones, 3rd- and 4th- generation cephalosporins, and colistin (the latter since January 1, 2025), through mandatory antimicrobial susceptibility testing (AST) (35). These regulations have contributed to more prudent antimicrobial use in veterinary medicine (36) and a reduced isolation of multi-resistant pathogens in dogs and cats (37, 38), including common bacterial isolates from the canine vagina (39).

Studies are needed to identify areas where antimicrobial use can be minimized. To date, no study has investigated microbiological sampling and antimicrobial treatment habits in canine reproductive medicine following new studies and legislation. It is hypothesized that antimicrobials are still being used with questionable indications in canine breeding. Therefore, this study aimed to assess the current veterinary practices in canine reproductive medicine in Germany to identify educational and research needs through a survey.

## 2 Materials and methods

Data were collected from May 25th, 2024 to November 28th, 2024 using an online questionnaire. The survey was conducted in German via the platform LimeSurvey® (Version 6.10.3, LimeSurvey GmbH, Hamburg, Germany). The target group comprised all small animal practitioners in Germany, as specified in a brief introduction providing relevant information about the survey. After consenting to the privacy policy, the questionnaire started, and responses were stored anonymously. Participants were recruited through the distribution of a link and a QR code. The survey was disseminated via e-mail distribution lists of veterinary associations (including various voluntary associations as well as the veterinary chambers of the federal states), personal contacts (e.g., at congresses, during veterinary courses, through personal networks, and in veterinary clinics and hospitals), and social media (particularly veterinary groups on Facebook). Additionally, the German Veterinary Journal, which is distributed to all registered veterinarians in Germany, was used to reach the target group.

The questionnaire included 37 questions, divided into five sections. Of these, 24 questions were dependent and were presented based on the selected answers. The survey contained single-choice (*n* = 27; SC) and multiple-choice (*n* = 9; MC) options, along with one free text entry (percentage), to ensure standardized responses for comparable analysis.

The initial part surveyed for fundamental information, including which species the participating veterinarian treated, work experience, current employment status, and whether they had dog breeders as clients. Part two outlined the general procedures of microbiological swab sampling and handling. It specified the frequency of sampling, the storage method, and the frequency of requesting AST. If swab samples were taken from the female reproductive tract, further questions were posed regarding the sampling frequency, indications, localizations, techniques, and culture examination conditions (aerobic/anaerobic). Furthermore, the survey inquired about the frequency of antimicrobial treatments requested by breeders prior to mating, as well as the rationales for these requests. Finally, a free text question assessed the proportion of samples taken from healthy bitches as part of breeding management examinations compared to those presumed to be diseased. The fourth section focused on the sampling of the male dog’s reproductive tract, similar to the bitch, encompassing aspects such as frequency, indications, culture examination conditions, as well as the frequency of, and reasons for, breeder-requested antimicrobial use before mating. The final part addressed the application of antimicrobial agents in the field of reproductive medicine in greater detail. It dealt with a quantification of male dog owners who insisted on antimicrobial treatment of the bitch before mating, covered the use of antimicrobials to treat reproductive diseases, the veterinarians’ practices and rationales for antimicrobial treatment prior to mating, and, in this context, the confidence in interpreting bacterial culture findings from the bitch’s genital flora. The entire questionnaire is presented in the Supplementary material.

With a population size of 10,652 (small animal practitioners in Germany in 2023) (40), a margin of error of 7%, and a confidence level of 95%, a minimum sample size of 197 participants was calculated for a representative sample size. For further data analysis only completed questionnaires of small animal practitioners were included. The saved data were exported to Microsoft Excel® (Version 2,503, Microsoft Corporation, Redmond, WA, USA). Data analysis was descriptive, and Microsoft Excel® was applied for the graphical presentation of the data. To determine whether a significant difference in antibiotic prescribing patterns prior to mating exist between participants with different work experiences, workplaces, or confidence levels, the chi-square test was carried out using GraphPad Prism, version 10.0.2 (GraphPad Software, Inc., Boston, MA, USA). Therefore, the participants’ work experience was classified into three categories: 1–5 years, 5–10 years, and more than 10 years. Workplace types were categoried into veterinary hospital or veterinary health center/referral clinic, large clinic, and small clinic, and confidence levels were grouped into confident and non-confident. A *p*-value of <0.05 was considered statistically significant.

## 3 Results

A total of 210 small animal veterinarians answered the survey completely, representing approximately 2% of all small animal practitioners in Germany (40).

### 3.1 Fundamental information on participants

**Figure 1:**
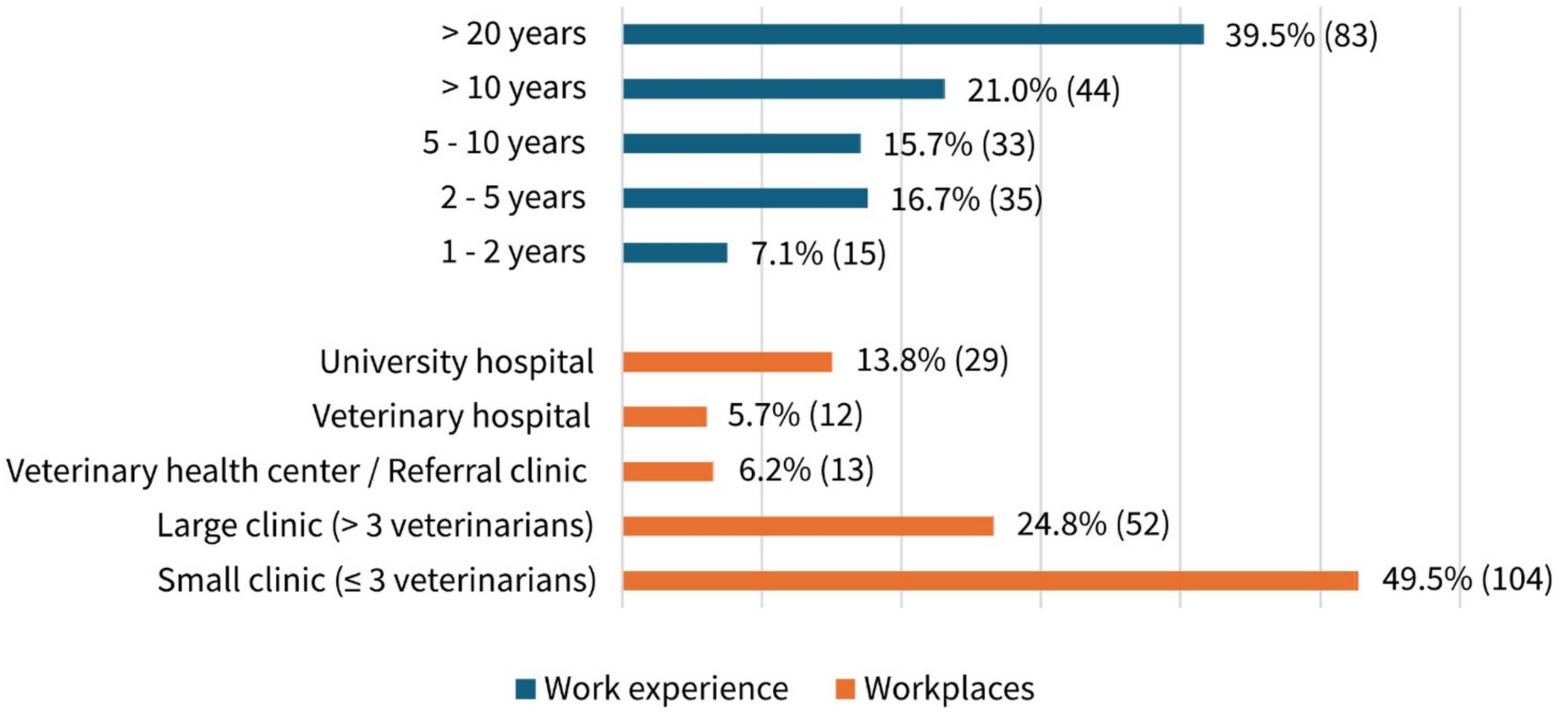
Work experience and workplaces of participants in percentage and absolute numbers. A total of 210 participants answered the question (single choice).

Almost 60% of participants (58.6%, 123/210, MC) reported exclusively treating small animals, while the remainder also treated other species, including livestock, horses, small mammals, exotics, and/or fish. The distribution between self-employed (51.4%, 108/210, SC) and employed veterinarians (47.2%, 99/210, SC) was almost equal, while 1.4% (3/210, SC) belonged to neither group (e.g., seeking employment). The work experience and workplaces are shown in Figure 1. More than half of the participants (60.5%, 127/210, SC) had been practicing for at least 10 years, with most working in small clinics (three or fewer veterinarians, 49.5%, 104/210, SC) (Figure 1).

The majority (78.6%, 165/210, SC) answered that they had dog breeders as clients, with most participants providing services to fewer than five breeders (41.0%, 86/210, SC). Nearly an equal proportion of participants served between five and 10 (17.6%, 37/210, SC) breeders, and more than 10 breeders (20.0%, 42/210, SC), respectively. In contrast, 21.4% (45/210, SC) of the participating veterinarians did not treat any dogs related to breeding purposes.

### 3.2 General procedures of microbiological swab sampling and handling

The most frequently cited indication for microbiological swab sampling in general was “If there is suspicion of a bacterial infection that requires antibiotics with mandatory AST” (66.7%, 140/210, SC). The second-ranked option was “If the initial therapy with antimicrobials is unsuccessful” (19.5%, 41/210, SC), followed by “Before every antimicrobial use” (12.9%, 27/210, SC). Two participants indicated that they never performed microbiological examinations (0.9%, 2/210, SC). The vast majority (82.7%, 172/208, SC) of those collecting samples for microbiology used a commercial laboratory for analysis. Other options included university laboratories (13.0%, 29/208, SC) and in-house laboratories (3.4%, 7/208, SC). In the majority of cases, samples were transported to the laboratory on a daily basis (88.0%, 183/208, SC). Conversely, in 12.0% (25/208, SC) of cases, samples were transported less frequently. Until further analysis, more than half of the participants stored the specimens under refrigeration (50.0%, 104/208, SC), while 38.5% (80/208, SC) stored them at room temperature. Moreover, in some cases, refrigerated storage was used in the event of high external temperatures (11.5%, 24/208, SC). The primary method of cooling was using a refrigerator (89.1%, 114/128, SC), but passive cooling with ice packs was also used occasionally (10.9%, 14/128, SC). Regarding the frequency of AST, 72.6% (151/208, SC) claimed to always request AST as part of microbiological examinations. Only 3.4% (7/208, SC) of respondents rarely requested AST, and none reported never requesting it.

### 3.3 Reproductive tract sampling and antimicrobial requests in bitches and male dogs

**Figure 2:**
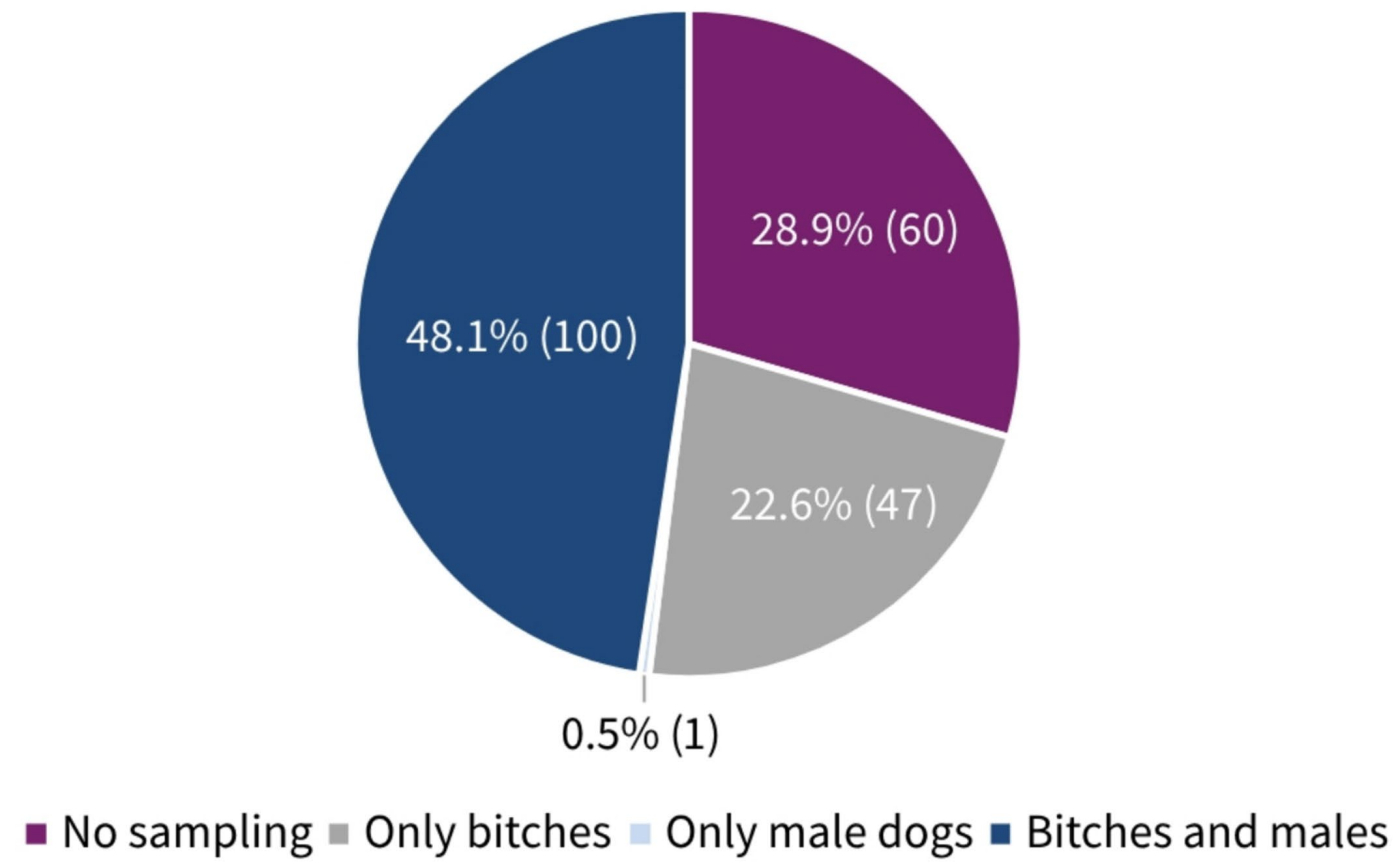
Percentage distribution and absolute numbers of participants performing reproductive tract sampling according to sexes. A total of 208 participants answered the question (single choice).

**Figure 3:**
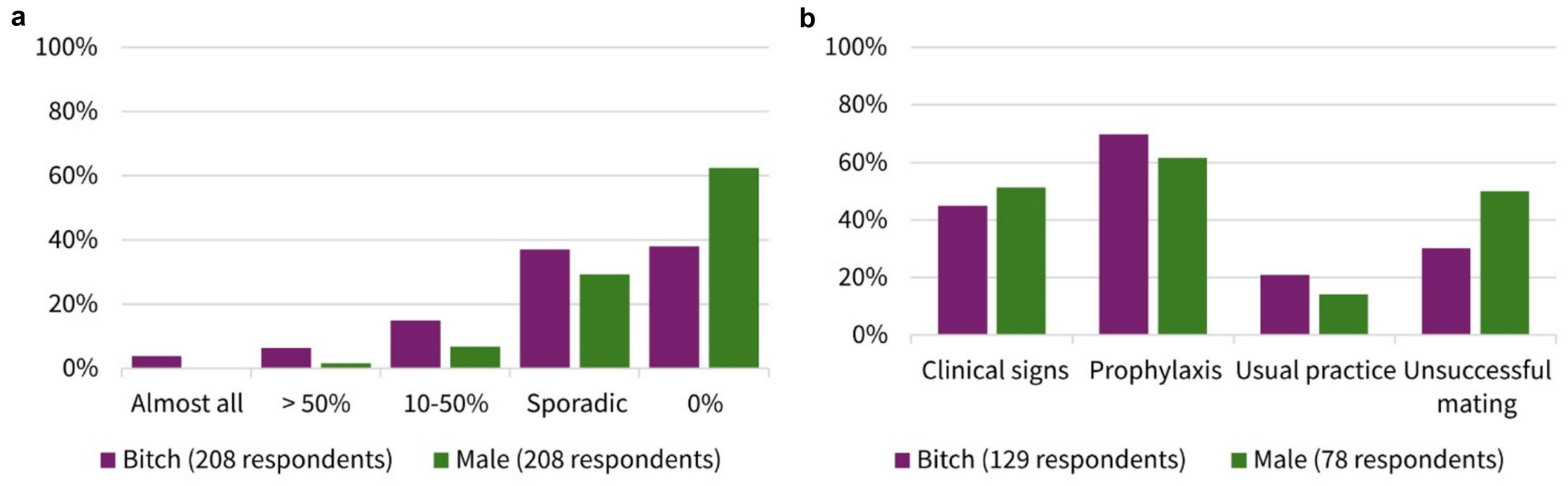
Comparative presentation of bitches and males for **(a)** frequency of breeders requesting antibiotic prescription prior to mating (single choice). **(b)** Breeders’ rationales for requesting antibiotics according to the surveyed veterinarians (multiple choice). Number of respondents dependent on the answer to previous question.

About 70% of the participants (70.1%, 147/208, SC) answered that they performed microbiological examinations from the female genital tract. In contrast to this, only 48.6% (101/208, SC) collected samples from the male genital tract (Figure 2). The participants’ frequency of sampling the reproductive tract is shown in Table 1. The main indications for collection in both genders (female vs. male) were suspicion of a bacterial infection (82.3%, 121/147 vs. 91.1%, 92/101, MC) and owner request (58.5%, 86/147 vs. 53.5%, 54/101, MC) (Table 1). Furthermore, 62.0% (129/208, SC) indicated that some breeders requested prescription of antimicrobials for their bitch prior to mating. One in 10 participants claimed that almost all (3.8%, 8/208, SC) or more than half (6.3%, 13/208, SC) of breeders requested this. Looking at the males, it is notable that still 37.5% (78/208, SC) of participants stated that antimicrobials were requested for male dogs before breeding. A higher number of respondents selected the answer option that no male dog owners asked for treatment prior to breeding, compared to owners of bitches (38.0%, 79/208 vs. 62.5%, 130/210, SC). The breeders’ rationales for requesting antimicrobials according to the surveyed veterinarians included (female vs. male) prophylaxis (69.8%, 90/129 vs. 61.5%, 48/78, MC), clinical signs of reproductive disease (45.0%, 58/129 vs. 51.3%, 40/78, MC), unsuccessful mating (30.2%, 39/129 vs. 50.0%, 39/79, MC), and/or usual practice (20.9%, 27/129 vs. 14.1%, 11/78, MC). All results are given in Figure 3.

**Table 1:** Number (*n*) and percentage (%) of responses regarding the frequency of reproductive tract sampling (single choice) and indications for sampling (multiple choice) in female/male dogs.

	*n* (Female/Male)	% (Female/Male)
Sampling frequency		
Very frequent (> 1 per week)	17 / 0	11.6 / 0
Frequent (> 2 per month)	17 / 9	11.6 / 8.9
Regular (1 per month)	31 / 19	21.1 / 18.8
Rare (< 1 per month)	82 / 73	55.7 / 72.3
Indications for sampling		
Suspicion of a bacterial infection	121 / 92	82.3 / 91.1
At owner’s request	86 / 54	58.5 / 53.5
Routinely as part of gynecologic/spermatologic examination	35 / 17	23.8 / 16.8

A total of 147 / 101 participants answered the question.

Whether aerobic and/or anaerobic culture conditions were requested was comparable for both sexes. The majority of respondents chose the conditions “depending on the clinical signs” (female vs. male: 46.9%, 69/147 vs. 52.5%, 53/101, SC). Infertility profiles offered by commercial laboratories were regularly used following conception failure (female vs. male: 55.1%, 81/147 vs. 48.5%, 49/101, SC). However, a substantial proportion of participants (female vs. male: 38.8%, 57/147 vs. 40.6%, 41/101, SC) also answered that they did not use these profiles at all.

### 3.4 Additional information about bitches

The most frequently sampled localizations of the female dog’s reproductive tract according to the participants’ answers were: 1. vagina (70.8%, 104/147, MC), 2. cervix (32.0%, 47/147, MC), 3. vestibule (21.1%, 31/147, MC), and 4. uterus (10.2%, 15/147, MC). The majority of participants generally used a speculum (72.8%, 107/147, SC), with the Kilian speculum (54.2%, 58/107, SC) being preferred compared to the tube speculum (45.8%, 49/107, SC). Only a small proportion of respondents applied other methods, such as intraoperative sampling (6.1%, 9/147, SC). Based on the participants’ answers regarding the proportion of samples taken from healthy bitches compared to those presumed diseased, the mean proportion was 44.4%.

### 3.5 Antimicrobial use in reproductive medicine

In terms of application of antimicrobial agents prior to mating, more than a third of respondents stated that male dog owners at least occasionally insisted on the bitch receiving antimicrobials prior to mating (36.7%, 77/210, SC); however, only very few participants answered that almost all (1.0%, 2/210, SC) or more than half (4.8%, 10/210, SC) of the owners of males requested this. In contrast, 30.9% (65/210, SC) answered that no male dog owner had this request, and 32.4% of participants (68/210, SC) were unable to provide a response.

Beta-lactam antibiotics were most commonly used for reproductive tract diseases, accounting for 83.8% (176/210, SC) of responses, followed by trimethoprim-sulfonamides with 8.1% (17/210, SC), fluoroquinolones with 4.3% (9/210, SC), and cephalosporins with 3.8% (8/210, SC). Antimicrobials were mainly administered for 5–7 days (68.6%, 144/210, SC). However, a total of 13.3% (28/210, SC) and 18.1% (38/210, SC) of the respondents treated shorter or longer, respectively.

**Figure 4:**
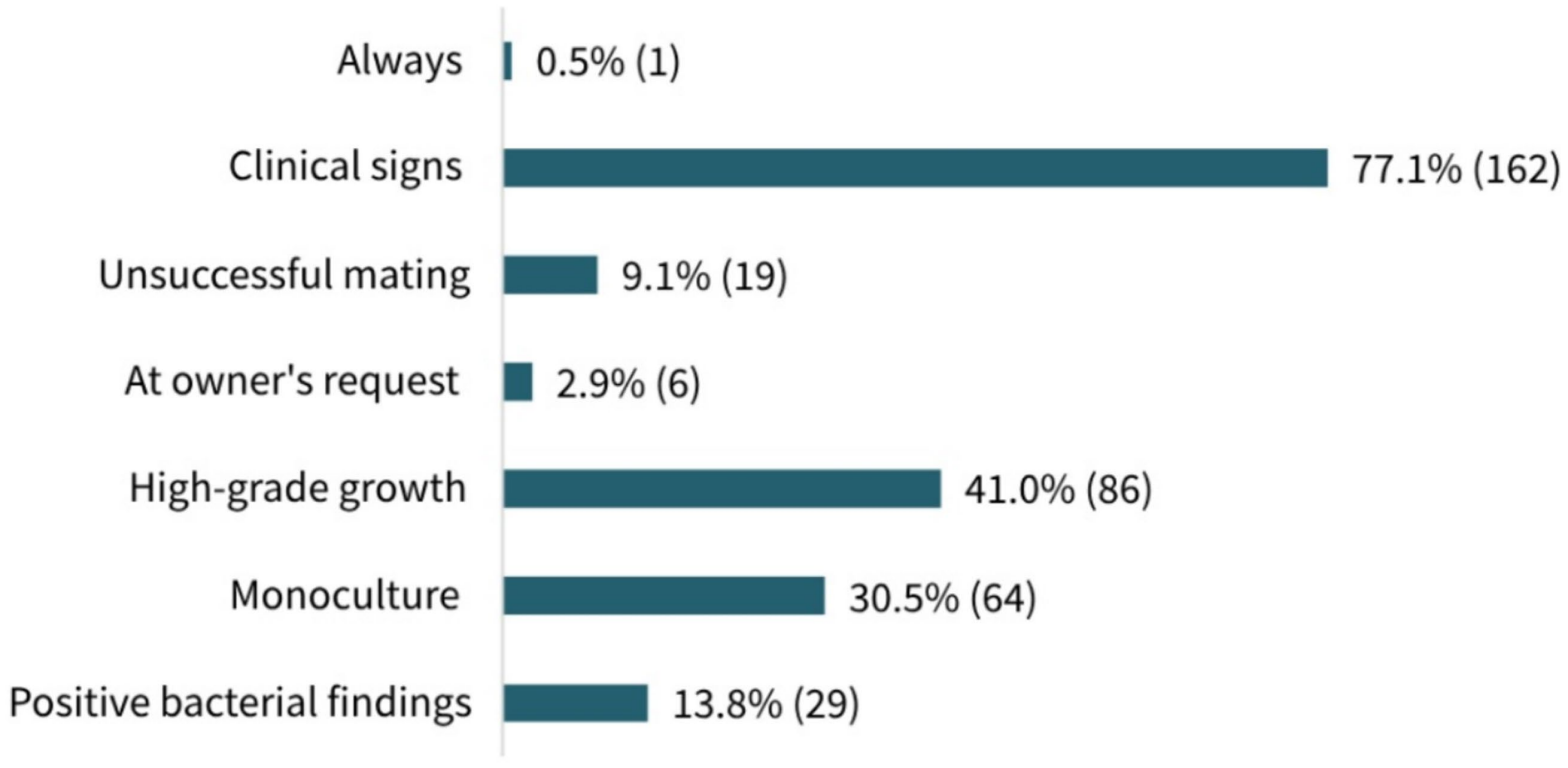
Participants’ rationales for antimicrobial use prior to mating. Results shown in percentage and absolute numbers of responses. A total of 210 participants answered the question (multiple choice).

The rationales for antimicrobial use prior to mating selected by participants are presented in Figure 4. The most common indication selected was “Presence of clinical signs in the reproductive tract” (77.1%, 162/210, MC), followed by “In case of high-grade bacterial growth” (41.0%, 86/213, MC) and “In case of bacterial monoculture” (30.5%, 64/210, MC). Nevertheless, 13.8% (29/210, MC) of the participants generally used antimicrobials if bacterial findings were positive and 9.1% (19/210, MC) stated that they applied antimicrobial agents if conception had failed in the previous cycle. The least frequent responses were “Always” (0.5%, 1/213, MC) and “At owner’s request” (2.9%, 6/210, MC). With respect to the participants’ work experience, no significant differences in prescribing patterns were observed between groups (*p* = 0.5527), and similarly, no significant differences were found between the workplace categories (*p* = 0.622).

The reasons for antimicrobial prescriptions at owner’s request were as follows: (1) to avoid confrontation/responsibility (4/6, MC), (2) whenever an AST is available (3/6, MC), (3) unsuccessful mating in previous cycle (2/6, MC), and (4) uncertainty about bacterial findings (2/6, MC). In these cases, the antimicrobial treatment was administered either orally (3/6, SC) or initially injected subcutaneously, followed by oral administration (3/6, SC).

**Figure 5:**
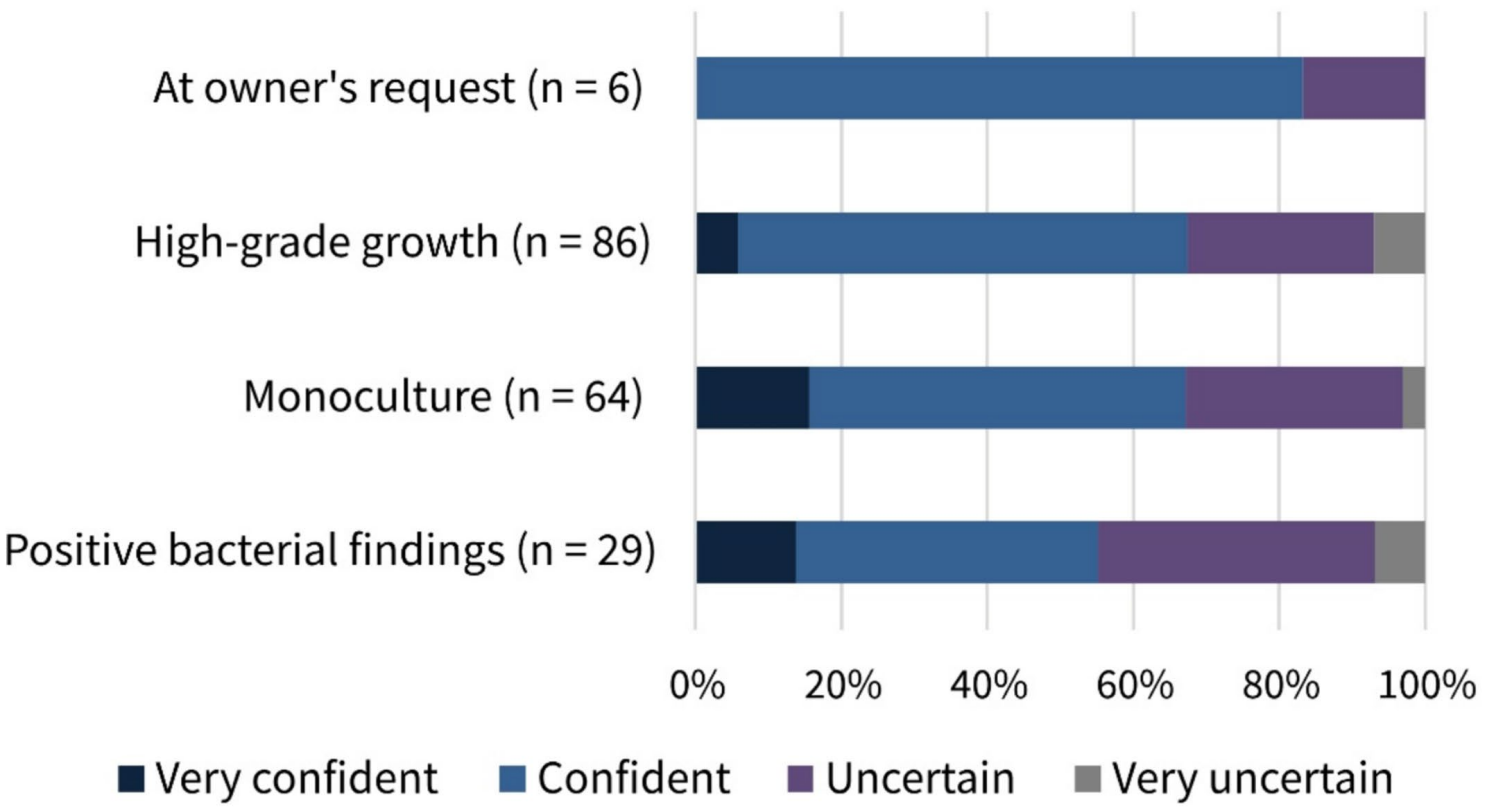
Selected rationales for antimicrobial use in healthy bitches prior to mating regarding participants’ confidence in interpreting bacterial culture findings. Number (*n*) of responses and percentage distribution (%) according to confidence.

The participants’ perception about their confidence in interpreting results of canine vaginal bacteriological examinations was mixed: whereas 57.6% (121/210, SC) felt confident or very confident, 42.4% (89/210, SC) were either uncertain or very uncertain (Table 2). Figure 5 shows the confidence of participants who administered antimicrobials to healthy bitches prior to breeding in certain cases. Overall, no significant differences were found in the rationales for prescribing antimicrobials before mating between participants who felt confident and those who felt uncertain (*p* = 0.7475).

**Table 2:** Number (*n*) and percentage (%) of responses regarding participants’ perception of their confidence in interpreting bacterial culture findings from the bitch’s genital flora.

	*n*	%
Participants’ confidence in interpreting bacterial culture findings		
Very uncertain	22	10.5
Uncertain	67	31.9
Confident	101	48.1
Very confident	20	9.5

A total of 210 participants answered the question (single choice).

Based on bacterial findings from swabs in the context of breeding management, 18.6% (39/210, SC) of respondents prescribed antimicrobials occasionally, 8.6% (18/210, SC) frequently, and 3.8% (8/210, SC) always. The majority rarely (31.9%, 67/210, SC) or never (37.1%, 78/210, SC) used antimicrobials in this regard.

After requesting an AST as part of breeding management, 53.8% (113/210, MC) of participants answered that they initiated antimicrobial treatment depending on the result, 34.8% (73/210, MC) claimed to wait for the AST, whereas 38.1% (80/210, MC) stated that they did not prescribe antimicrobials without any clinical signs regardless of the findings. Before receiving the results of bacterial culture and AST, some respondents answered that they occasionally started with HIA/CIA (15.2%, 32/210, MC) and HPCIA (3.8%, 8/210, MC), respectively.

## 4 Discussion

To our knowledge, this is the first investigation into habits of German veterinarians regarding microbiological sampling and antimicrobial treatment practices in canine reproductive medicine following recent legislative developments. A survey from a 2008 thesis confirmed that vaginal swab sampling and antimicrobial use before mating are common practice among German veterinarians and breeders (30); no updated data have been published since then.

Our study confirms that, as anticipated, a large proportion of the vaginal swabs collected in veterinary practice originate from clinically healthy dogs. Among participants who sampled the female reproductive tract, nearly half of the swabs were collected in the context of breeding management. In general, this is not problematic; however, swab results frequently result in antimicrobial treatment, or at least in the request for such. This habit is questionable, as several culture-based studies on the canine vaginal flora have emphasized that there is no difference in bacterial composition between healthy animals and those with reproductive diseases (12–17). Bacterial culture examination is therefore of limited diagnostic value in the absence of clinical symptoms such as abnormal vaginal discharge. Although culture-independent methods can also provide a more comprehensive picture of the microbiota in the female dogs’ reproductive tract, including hard-to-culture organisms (18–20), they are currently not a viable alternative to established culture-based diagnostics in clinical practice due to limitations in availability, costs, and the possibility of AST, but also difficulties in interpretation of the results. Analysis of the reasons for bacteriological examination of the genital tract revealed that owner request was a major factor, selected by over 50% of respondents. This highlights the importance of breeder education and effective communication by the attending veterinarian regarding the limited diagnostic value and questionable benefit of such examinations. This is essential to reduce unreasonable antimicrobial use in canine breeding in healthy bitches.

Bertero et al. pointed out that dogs from breeding kennels in Italy had higher AMR rates compared to household animals (41). This indicates that, in agreement with other studies, canine breeding is of particular concern for AMR development and transmission, suggesting that overuse and misuse of antimicrobials prior to mating is common practice (31–33, 41). Despite the lack of specific data from German kennels and breeders, our results further support this hypothesis, as 62% of the surveyed veterinarians reported that some breeders requested antimicrobial prescription for their bitches. According to the participating veterinarians, breeders primarily requested antimicrobials for prophylactic use or usual practice, once again underlining the importance of education and communication with breeders to prevent antimicrobial misuse. Although breeders might play a more important role because of larger numbers of dogs kept per kennel and household, many pet owners also lack knowledge about antimicrobials and the development and consequences of AMR (21, 42, 43). Wright et al. demonstrated that even a brief educational animation can raise owners’ awareness of antimicrobial use (44). Therefore, clear and transparent communication is key. Beside direct communication with the attending veterinarian, easily accessible resources such as animated clips and webinars could be helpful. Furthermore, breeder seminars and journals represent valuable channels that should be utilized to disseminate information through lectures and articles.

Another frequently selected rationale for antimicrobial treatment was unsuccessful mating in a previous cycle. Considering the numerous potential causes of conception failure (45, 46), there is not only a risk of missing the underlying reason for subfertility/infertility, but also of disrupting the balance of the reproductive flora. Various studies in women have emphasized the importance of a balanced vaginal microbiome for reproductive health (47–49). The administration of antimicrobial agents can lead to dysbiosis by eliminating competitive bacteria, potentially promoting the overgrowth of opportunistic bacteria (e.g., *E. coli* and *Sc. canis*) (6, 17, 50). This not only contributes to AMR development in these pathogens but has also been shown to impair fertility and genital health (6, 10–12, 17).

Besides clinical signs of reproductive tract diseases, antimicrobial use prior to mating in cases of high-grade bacterial growth and bacterial monoculture was commonly reported by the surveyed veterinarians; however, this is contradictory to recommendations made in recent studies. According to Schäfer-Somi et al. and Leps et al., based on large study populations, antimicrobials should not be administered in the absence of clinical signs, even in cases of high-grade monocultures (8, 9). Our findings highlight the need to also educate veterinarians, especially those who are not well informed about recent developments in the field of canine reproductive medicine. A large proportion of the participants worked in small clinics, where comprehensive veterinary knowledge is required. However, no significant differences in the prescribing patterns were observed between different workplaces or work experiences of participants. Thus, using various channels to reach a broad spectrum of the veterinary profession, along with easy access to essential findings through evidence-based guidelines and continuing education, represents an important tool, particularly in the field of small animal reproduction, a niche area of growing interest (51). In addition to traditional forms of continuing education, such as lectures and journal articles, easily accessible (online) resources like webinars should be used, just as for breeders.

Although it has been known for a long time that the canine vagina is not a sterile environment (3–9), our results support the hypothesis that many breeders and some veterinarians still associate the presence of vaginal bacteria with infection and infertility. The fact that 13.6% of participating veterinarians stated that they regularly administered antimicrobials if bacterial findings were positive, once again highlights the need for further education, since no bacterial growth in vaginal swab cultures is physiologically rare and could be indicative of inadequate sampling and/or culturing (6). Moreover, it is concerning that antimicrobials are prescribed at the owner’s request. When asked why, four of six respondents indicated that they did so to avoid confrontation. This suggests that veterinarians may sometimes experience pressure from breeders, which agrees with findings from other survey studies (21, 43, 52).

More than 50% of the participants reported feeling confident or very confident about interpreting bacterial culture findings from the bitch’s genital flora. Nevertheless, nearly 50% stated feeling uncertain or very uncertain when determining the need for antimicrobial treatment, additionally indicating the urgent need for veterinary support and education. Interestingly, Figure 5 shows that most of the veterinarians who reported using antimicrobials in healthy bitches in certain cases, in contrast to recent recommendations, indicated feeling confident or even very confident when doing so. Furthermore, prescribing patterns prior to mating did not differ significantly between participants’ confidence levels. This highlights the importance of continuous veterinary education, as medicine is constantly evolving. Findings that introduce new approaches challenging established habits should be disseminated with the support of recognized key opinion leaders in order to build trust and promote continuous improvement of usual practices.

The study data showed that participants sampled from different sites using different sampling techniques. This is potentially problematic, as sampling from the caudal vagina or vestibule without the use of a speculum could falsify the results (4, 15, 53) and lead to an unreasonable antimicrobial use, especially when bacterial quantity is used as the basis for treatment decisions. Furthermore, factors such as sample storage, shipment, and processing as well as storage temperature affect the microbiological outcome (54, 55). Nevertheless, the heterogeneity of responses provided by the participants reflects the need for further research into sample handling, as standardized procedures are essential to ensure reliable and comparable interpretation of swab examinations.

Legislative restrictions and guidelines have been shown to contribute to more prudent antimicrobial use and greater awareness (36, 56, 57). For instance, the implementation of the TÄHAV was generally associated with reduced AMR rates and increased AST (36–38), also in the reproductive tract (39). Nevertheless, one important finding was that most participants answered that they only performed microbiological swab sampling in cases of suspected bacterial infection requiring antibiotics with mandatory AST, or when initial antimicrobial therapy failed. Given that only 12.9% of respondents reported performing microbiological testing before every antimicrobial use, it is strongly advised to implement regular AST. This approach not only improves treatment efficacy but also helps prevent AMR (22). On the other hand, mandatory AST might result in even more frequent unnecessary antimicrobial treatments, as the presence of ASTs might justify antimicrobial treatment or even indicate the need for treatment in the case of veterinarians being uncertain about the interpretation or breeders urging for treatment because an AMR was made by the laboratory because of presumed pathogenicity. Therefore, we recommend that the TÄHAV should be revised for more prudent use of antimicrobials, as, except for certain HPCIA, it currently permits the administration of antimicrobials in companion animals without regulation. Moreover, it only authorizes AST for HPCIA, without addressing the implications of the test results for subsequent treatment decisions (35).

Furthermore, a joint global action plan by the WHO, the Food and Agriculture Organization (FAO), and the World Organization for Animal Health (OIE) emphasized the importance of surveillance for risk assessment, enabling the identification of areas where targeted interventions are required (26). From 2026 onwards, mandatory reporting of antimicrobial use in Germany will also include dogs and cats in addition to livestock species (58). These data should be included for future studies on antimicrobial prescribing patterns.

The major limitation of our study was that participation was voluntary, which may have resulted in an overrepresentation of participants with a greater interest in the topic and a higher confidence in their responses. In addition, the response options were predetermined, which was necessary for a comparable analysis. Although multiple channels (e-mail lists, personal contacts, social media, and the German Veterinary Journal) were used to reach small animal practitioners in Germany, the distribution may have reached only a selected subgroup of the target population. Veterinarians who are more engaged in professional networks, continuing education, or online platforms may have been more likely to notice and respond to the survey, while those less digitally connected or less active in professional circles may be underrepresented. Furthermore, the use of personal contacts could have also resulted in clustering within certain professional or regional networks with shared interests.

The strength of our study lies in the participation of approximately 2% of all small animal practitioners in Germany, fulfilling the calculated minimum sample size. Moreover, the used various channels allowed for wide dissemination, and official channels such as the German Veterinary Journal and the regional veterinary chambers ensured that, in principle, the entire target group had the opportunity to participate. Therefore, the risk of systematic exclusion of specific subgroups is considered limited. The demographic composition of participants closely mirrors the population of small animal practitioners in Germany (40), supporting the representativeness of our results.

## 5 Conclusion

This study reveals that antimicrobial use in clinically healthy breeding bitches is still common practice, often driven by routine procedures and owner requests. Our findings highlight the importance of targeted education for veterinarians and improved communication with breeders to promote rational antimicrobial use, enabling them to reflect on, and improve, their management practices, on a continuous basis. Therefore, easy access to proper instructions is essential. Future tools could include webinars, animated clips, articles of international key opinion leaders in veterinary and breeder journals, and lectures for the respective groups. Future research should focus on the reproductive microbiome in relation to fertility to allow for evidence-based decisions regarding the interpretation of vaginal bacterial findings and valid indications for antimicrobial treatment. Finally, we should critically evaluate each administration to ensure alignment with antimicrobial stewardship principles, thus preserving the efficacy of available treatments and reducing the risk of antimicrobial resistance in the long term.

## Data Availability

The raw data supporting the conclusions of this article will be made available by the authors, without undue reservation.

## References

[1] Ravel J Gajer P Abdo Z Schneider GM Koenig SS McCulle SL. Vaginal microbiome of reproductive-age women. Proc Natl Acad Sci USA. (2011) 108:4680–7. doi: 10.1073/pnas.1002611107, PMID: 20534435 PMC3063603

[2] Verstraelen H Vieira-Baptista P De Seta F Ventolini G Lonnee-Hoffmann R Lev-Sagie A. The vaginal microbiome: I. Research development, lexicon, defining "Normal" and the dynamics throughout women's lives. J Low Genit Tract Dis. (2022) 26:73–8. doi: 10.1097/LGT.0000000000000643, PMID: 34928256 PMC8719517

[3] Bjurstrom L Linde-Forsberg C. Long-term study of aerobic Bacteria of the genital tract in breeding bitches. Am J Vet Res. (1992) 53:665–9. doi: 10.2460/ajvr.1992.53.05.665, PMID: 1524290

[4] Olson PN Mather EC. Canine vaginal and uterine bacterial flora. J Am Vet Med Assoc. (1978) 172:708–10. 640936

[5] Watts JR Wright PJ Whithear KC. Uterine, cervical and vaginal microflora of the Normal bitch throughout the reproductive cycle. J Small Anim Pract. (1996) 37:54–60. doi: 10.1111/j.1748-5827.1996.tb01936.x, PMID: 8656593

[6] Groppetti D Pecile A Barbero C Martino PA. Vaginal bacterial Flora and Cytology in Proestrous bitches: role on fertility. Theriogenology. (2012) 77:1549–56. doi: 10.1016/j.theriogenology.2011.11.022, PMID: 22289216

[7] Maksimovic A Maksimovic Z Filipovic S Besirovic H Rifatbegovic M. Vaginal and uterine bacteria of healthy bitches during different stages of their reproductive cycle. Vet Rec. (2012) 171:375. doi: 10.1136/vr.100886 22903902

[8] Schäfer-Somi S Lechner D Tichy A Spergser J. The cultivable Bacteria colonizing canine vagina during Proestrus and estrus: a large-scale retrospective study of influencing factors. Animals. (2024) 14:3460. doi: 10.3390/ani14233460, PMID: 39682423 PMC11640309

[9] Leps AS Klein B Schneider M Meyer C Soba A Simon C. The canine vaginal Flora: a large-cohort retrospective study. Vet Sci. (2024) 11:55. doi: 10.3390/vetsci11020055, PMID: 38393073 PMC10892940

[10] Shambulingappa BE Manegar GA Ananda KJ. Study on aerobic bacterial flora in canine abortions. Vet World. (2010) 3:111–2.

[11] Graham EM Taylor DJ. Bacterial reproductive pathogens of cats and dogs. Vet Clin North Am Small Anim Pract. (2012) 42:561–82. doi: 10.1016/j.cvsm.2012.01.013, PMID: 22482819

[12] Bjurstrom L. Aerobic Bacteria occurring in the vagina of bitches with reproductive disorders. Acta Vet Scand. (1993) 34:29–34. doi: 10.1186/BF03548220, PMID: 8342462 PMC8112530

[13] Osbaldiston GW Nuru S Mosiert JE. Vaginal cytology and microflora of intertile bitches. J Am Anim Hosp Assoc. (1972) 8:93–101.

[14] Hirsh DC Wiger N. The bacterial Flora of the Normal canine vagina compared with that of vaginal exudates. J Small Anim Pract. (1977) 18:25–30. doi: 10.1111/j.1748-5827.1977.tb05820.x, PMID: 853729

[15] Allen Edward W Dagnall GJR. Some observations on the aerobic bacterial flora of the genital tract of the dog and bitch. J Small Anim Pract. (1982) 23:325–35. doi: 10.1111/j.1748-5827.1982.tb01674.x

[16] Golinska E Sowinska N Tomusiak-Plebanek A Szydlo M Witka N Lenarczyk J. The vaginal microflora changes in various stages of the estrous cycle of healthy female dogs and the ones with genital tract infections. BMC Vet Res. (2021) 17:8. doi: 10.1186/s12917-020-02710-y, PMID: 33407480 PMC7789644

[17] Jagodka D Kaczorek-Lukowska E Graczyk R Socha P. Vaginal aerobic bacteria of healthy bitches and those with fertility problems. Pol J Vet Sci. (2023) 26:733–9. doi: 10.24425/pjvs.2023.148293 38088743

[18] Rota A Corro M Patuzzi I Milani C Masia S Mastrorilli E. Effect of sterilization on the canine vaginal microbiota: a pilot study. BMC Vet Res. (2020) 16:455. doi: 10.1186/s12917-020-02670-3, PMID: 33228646 PMC7684734

[19] Lyman CC Holyoak GR Meinkoth K Wieneke X Chillemi KA DeSilva U. Canine endometrial and vaginal microbiomes reveal distinct and complex ecosystems. PLoS One. (2019) 14:e0210157. doi: 10.1371/journal.pone.0210157, PMID: 30615657 PMC6322750

[20] Leps AS Packeiser E-M Schwens C Stoelcker B Doric S Wirkner M. The canine vaginal microbiome during heat and fertility in healthy breeding dogs. PLoS One. (2025) 20:e0321683. doi: 10.1371/journal.pone.0321683, PMID: 40293987 PMC12036845

[21] Cazer CL Lawless JW Frye A Gonzalez L Greiner SA. Divergent veterinarian and cat owner perspectives are barriers to reducing the use of Cefovecin in cats. J Am Vet Med Assoc. (2023) 261:1810–9. doi: 10.2460/javma.23.08.0487, PMID: 37918112

[22] Weese JS Giguere S Guardabassi L Morley PS Papich M Ricciuto DR. Acvim consensus statement on therapeutic antimicrobial use in animals and antimicrobial resistance. J Vet Intern Med. (2015) 29:487–98. doi: 10.1111/jvim.12562, PMID: 25783842 PMC4895515

[23] World Health Organization. (2023) Antimicrobial resistance. Available online at: https://www.who.int/news-room/fact-sheets/detail/antimicrobial-resistance. [Accessed March 31, 2025]

[24] Christaki E Marcou M Tofarides A. Antimicrobial resistance in Bacteria: mechanisms, evolution, and persistence. J Mol Evol. (2020) 88:26–40. doi: 10.1007/s00239-019-09914-3, PMID: 31659373

[25] Lee JH. Perspectives towards antibiotic resistance: from molecules to population. J Microbiol. (2019) 57:181–4. doi: 10.1007/s12275-019-0718-8, PMID: 30806975

[26] Guardabassi L Butaye P Dockrell DH Ross Fitzgerald J Kuijper EJ. One health: a multifaceted concept combining diverse approaches to prevent and control antimicrobial resistance. Clin Microbiol Infect. (2020) 26:1604–5. doi: 10.1016/j.cmi.2020.07.012 32702500

[27] McEwen SA Collignon PJ. Antimicrobial resistance: a one health perspective. Microbiol Spectr. (2018) 6:2017. doi: 10.1128/microbiolspec.ARBA-0009-2017 PMC11633550 29600770

[28] Pomba C Rantala M Greko C Baptiste KE Catry B van Duijkeren E. Public health risk of antimicrobial resistance transfer from companion animals. J Antimicrob Chemother. (2017) 72:957–68. doi: 10.1093/jac/dkw481, PMID: 27999066

[29] Guardabassi L Schwarz S Lloyd DH. Pet animals as reservoirs of antimicrobial-resistant bacteria. J Antimicrob Chemother. (2004) 54:321–32. doi: 10.1093/jac/dkh332 15254022

[30] Jeschke T. Erhebung Zur Situation Der Caninen Reproduktionsmedizin Bei Tierärzten Und Züchtern - Ein Beitrag Zur Erhebung Des Status Quo Und Zur Verbesserung Der Lehre Auf Diesem Gebiet. Germany: Justus-Liebig-Universität Gießen (2008).

[31] Rota A Milani C Drigo I Drigo M Corro M. Isolation of methicillin-resistant *Staphylococcus Pseudintermedius* from breeding dogs. Theriogenology. (2011) 75:115–21. doi: 10.1016/j.theriogenology.2010.07.016, PMID: 20961604

[32] Milani C Corro M Drigo M Rota A. Antimicrobial resistance in Bacteria from breeding dogs housed in kennels with differing neonatal mortality and use of antibiotics. Theriogenology. (2012) 78:1321–8. doi: 10.1016/j.theriogenology.2012.05.033, PMID: 22898018

[33] Rota A Milani C Corro M Drigo I Borjesson S. Misuse of antimicrobials and selection of methicillin-resistant *Staphylococcus pseudintermedius* strains in breeding kennels: genetic characterization of bacteria after a two-year interval. Reprod Domest Anim. (2013) 48:1–6. doi: 10.1111/j.1439-0531.2012.02012.x, PMID: 22551469

[34] World Health Organization. (2024) Who's list of medically important antimicrobials: A risk management tool for mitigating antimicrobial resistance due to non-human use. Available online at: https://cdn.who.int/media/docs/default-source/gcp/who-mia-list-2024-lv.pdf?sfvrsn=3320dd3d_2. [Accessed March 31, 2025]

[35] Tierärztliche-Hausapothekenverordnung. (2024) Verordnung Über Tierärztliche Hausapotheken (TÄHAV). Available online at: https://www.gesetze-im-internet.de/t_hav_2025/BJNR1570A0024.html. [Accessed March 31, 2025]

[36] Moerer M Merle R Bäumer W. A cross-sectional study of veterinarians in Germany on the impact of the Tahav amendment 2018 on antimicrobial use and development of antimicrobial resistance in dogs and cats. Antibiotics. (2022) 11:484. doi: 10.3390/antibiotics11040484, PMID: 35453235 PMC9028039

[37] Loeffler A Beever L Chang Y-M Klein B Kostka V Meyer C. Intervention with impact: reduced isolation of methicillin-resistant *Staphylococcus Pseudintermedius* from dogs following the introduction of antimicrobial prescribing legislation in Germany. Vet Record. (2024) 194:e3714. doi: 10.1002/vetr.3714, PMID: 38100180

[38] Moerer M Lubke-Becker A Bethe A Merle R Bäumer W. Occurrence of antimicrobial resistance in canine and feline bacterial pathogens in Germany under the impact of the Tahav amendment in 2018. Antibiotics. (2023) 12:1193. doi: 10.3390/antibiotics12071193, PMID: 37508289 PMC10376885

[39] Leps AS Klein B Schneider M Goericke-Pesch S. How restrictive legislation influences antimicrobial susceptibility in selected bacterial isolates from the canine vagina. Antibiotics. (2024) 13:946. doi: 10.3390/antibiotics13100946, PMID: 39452212 PMC11504881

[40] Bundestierärztekammer. (2023) Tierärztestatistik 2023. Available online at: https://www.bundestieraerztekammer.de/btk/statistik/. [Accessed March 23, 2025]

[41] Bertero A Corro M Del Carro A Spagnolo E Milani C Diana A. Antimicrobial pressure in healthy breeding dogs vs household animals assessed through the resistance profile of Escherichia Coli and coagulase positive staphylococci. Vet J. (2025) 311:106337. doi: 10.1016/j.tvjl.2025.106337, PMID: 40120715

[42] Frey E Kedrowicz A Hedgpeth M-W. Decision making on antimicrobial use: cat and dog owners' knowledge and preferences for veterinary communication. Vet Record. (2024) 194:e3411. doi: 10.1002/vetr.3411, PMID: 37691448

[43] Smith M King C Davis M Dickson A Park J Smith F. Pet owner and vet interactions: exploring the drivers of AMR. Antimicrob Resist Infect Control. (2018) 7:46. doi: 10.1186/s13756-018-0341-1 29619213 PMC5879597

[44] Wright E Jessen LR Tompson A Rutland C Singleton D Battersby I. Influencing attitudes towards antimicrobial use and resistance in companion animals-the impact on pet owners of a short animation in a randomized controlled trial. JAC Antimicrob Resist. (2024) 6:dlae065. doi: 10.1093/jacamr/dlae065, PMID: 38716404 PMC11073752

[45] Wilborn RR Maxwell HS. Clinical approaches to infertility in the bitch. Vet Clin North Am Small Anim Pract. (2012) 42:457–68. doi: 10.1016/j.cvsm.2012.01.016, PMID: 22482812

[46] Mantziaras G Zakosek Pipan M. "My bitch is empty!" an overview of the reasons for pregnancy loss in dogs. Vet Sci. (2025) 12:127. doi: 10.3390/vetsci12020127, PMID: 40005887 PMC11860774

[47] Chen X Lu Y Chen T Li R. The female vaginal microbiome in health and bacterial vaginosis. Front Cell Infect Microbiol. (2021) 11:631972. doi: 10.3389/fcimb.2021.631972, PMID: 33898328 PMC8058480

[48] Saraf VS Sheikh SA Ahmad A Gillevet PM Bokhari H Javed S. Vaginal microbiome: normalcy vs dysbiosis. Arch Microbiol. (2021) 203:3793–802. doi: 10.1007/s00203-021-02414-3, PMID: 34120200

[49] Ughade PA Shrivastava D Chaudhari K. Navigating the microbial landscape: understanding dysbiosis in human genital tracts and its impact on fertility. Cureus. (2024) 16:e67040. doi: 10.7759/cureus.67040 39286717 PMC11403153

[50] Ström B Linde-Forsberg C. Effects of ampicillin and trimethoprim-sulfamethoxazole on the vaginal bacterial Flora of bitches. Am J Vet Res. (1993) 54:891–6. doi: 10.2460/ajvr.1993.54.06.891, PMID: 8323058

[51] Fontbonne A. Small animal reproduction: scientific facts versus dogmas or unverified beliefs. Theriogenology. (2020) 150:464–70. doi: 10.1016/j.theriogenology.2020.03.014, PMID: 32284211 PMC7102635

[52] Hopman NEM Hulscher M Graveland H Speksnijder DC Wagenaar JA Broens EM. Factors influencing antimicrobial prescribing by Dutch companion animal veterinarians: a qualitative study. Prev Vet Med. (2018) 158:106–13. doi: 10.1016/j.prevetmed.2018.07.013, PMID: 30220383

[53] Root Kustritz MV. Collection of tissue and culture samples from the canine reproductive tract. Theriogenology. (2006) 66:567–74. doi: 10.1016/j.theriogenology.2006.05.003 16750845

[54] Poulsen CS Kaas RS Aarestrup FM Pamp SJ. Standard sample storage conditions have an impact on inferred microbiome composition and antimicrobial resistance patterns. Microbiol Spectr. (2021) 9:e0138721. doi: 10.1128/Spectrum.01387-21, PMID: 34612701 PMC8510183

[55] Panisello Yagüe D Mihaljevic J Mbegbu M Wood CV Hepp C Kyman S. Survival of *Staphylococcus Aureus* on sampling swabs stored at different temperatures. J Appl Microbiol. (2021) 131:1030–8. doi: 10.1111/jam.15023, PMID: 33544965 PMC8339145

[56] Jessen LR Sorensen TM Lilja ZL Kristensen M Hald T Damborg P. Cross-sectional survey on the use and impact of the Danish National Antibiotic use Guidelines for companion animal practice. Acta Vet Scand. (2017) 59:81. doi: 10.1186/s13028-017-0350-8 29228960 PMC5725655

[57] Hopman NEM Portengen L Hulscher M Heederik DJJ Verheij TJM Wagenaar JA. Implementation and evaluation of an antimicrobial stewardship programme in companion animal clinics: a stepped-wedge design intervention study. PLoS One. (2019) 14:e0225124. doi: 10.1371/journal.pone.0225124, PMID: 31738811 PMC6860428

[58] Tierarzneimittelgesetz. (2021) Gesetz Über Den Verkehr Mit Tierarzneimitteln Und Zur Durchführung Unionsrechtlicher Vorschriften Betreffend Tierarzneimittel (TAMG). Available online at: https://www.gesetze-im-internet.de/tamg/. [Accessed March 31, 2025]

