# Supplementary material for: German veterinarians asked: a cross-sectional study on microbiological examination and antimicrobial use in canine reproductive medicine
Source: Front Vet Sci. 2025 Jul 29;12:1645496. doi: 10.3389/fvets.2025.1645496 (PMC12339326; doi:10.3389/fvets.2025.1645496)
Supplement: Supplementary file 1 [file Data_Sheet_1.PDF]

Supplementary Material

1 Supplementary Data

Questionnaire:

1. Which animal species do you treat? (multiple choice)

a. Small animals

b. Livestock

c. Horses

d. Small mammals and exotics

e. Fish

2. How long have you been practicing? (single choice)

a. 1–2 years

b. 2–5 years

c. 5–10 years

d. More than 10 years

e. More than 20 years

3. Are you self-employed or employed? (single choice)

a. Self-employed

b. Employed

c. Other (e.g., seeking employment)

4. Do you work in a clinic or a hospital? (single choice)

a. Small clinic (≤ 3 veterinarians)

b. Large clinic (> 3 veterinarians)

c. Veterinary health center/Referral clinic

d. Veterinary hospital

e. University hospital

5. Do you have dog breeders as clients? If yes, how many? (single choice)

a. No

b. Yes, fewer than 5

c. Yes, between 5 and 10

d. Yes, more than 10

6. How often do you collect swab samples for microbiological examination in dogs? (single choice)

a. Before every antimicrobial use

b. If there is suspicion of a bacterial infection that requires antibiotics with mandatory antimicrobial susceptibility testing

c. If the initial therapy with antimicrobials is unsuccessful

d. Never

Follow-up questions if options 6 a, b, c are selected. Microbiological swab sampling (6.1 – 6.7):

6.1. Where are the collected samples analyzed? (single choice)

a. Commercial laboratory

b. University facility

c. In-house laboratory

6.2 How regularly are the samples transported to the laboratory? (single choice)

a. Daily

b. Less frequently

6.3 How do you store the samples until transport or processing? (single choice)

a. At room temperature

b. Refrigerated

c. Only refrigerated in the event of high external temperatures (>25 °C)

Follow-up question if options 6.3 b, c are selected.

At what temperature do you store the samples? (single choice)

a. Actively refrigerated (4 °–8 °C)

b. Passively cooled (e.g., Styrofoam box with ice packs)

6.4 How often do you request antimicrobial susceptibility testing? (single choice)

a. Always

b. Usually

c. Sometimes

d. Rarely (only if deemed necessary)

e. Never

6.5 Do you collect swab samples from the reproductive tract of dogs? (single choice)

a. Only bitches

b. Only male dogs

c. Bitches and males

d. No

Follow-up questions if options 6.5 a, c are selected. Questions regarding the bitch (6.5.1):

6.5.1.1 How often do you collect swabs from the female reproductive tract? (single choice)

a. Very frequently (more than once per week)

b. Frequently (more than twice per month)

c. Regularly (once per month)

d. Rarely (less than once per month)

6.5.1.2 When do you take swabs from the female reproductive tract? (multiple choice)

a. Suspicion of a bacterial infection

b. Routinely as part of gynecologic examination

c. At owner's/breeder's request

6.5.1.3 Which site do you take samples from in the female reproductive tract? (multiple choice)

a. Vestibule

b. Vagina

c. Cervix

d. Uterus

6.5.1.4 What is your preferred sampling method? (single choice)

a. Without speculum

b. With speculum

c. Other (e.g., intraoperative sampling)

Follow-up question if option 6.5.1.4 b is selected.

Which speculum model do you use? (single choice)

a. Tube speculum (Hannover model)

b. Kilian speculum

6.5.1.5 Which culture conditions do you request? (single choice)

a. Aerobic only

b. Aerobic and anaerobic

c. Varies depending on the clinical signs

6.6 How many breeders/owners request antimicrobial prescription for their bitch prior to mating? (single choice)

a. Very many (almost all)

b. Many (more than half)

c. Some (10–50%)

d. Few (sporadic)

e. None

Follow-up question if options 6.6 a, b, c, d are selected.

What are the breeders' rationales for requesting antimicrobial prescription prior to mating? (multiple choice)

a. Clinical signs of reproductive disease

b. Unsuccessful mating in previous cycle

c. Prophylaxis

d. Usual practice

6.5.1.6 Do you use commercial "infertility profiles" (e.g., CHV-PCR, Mycoplasma spp.-PCR, Chlamydia spp.-PCR)? (single choice)

a. Always

b. Regularly

c. In case of unsuccessful previous mating

d. Never

6.5.1.7 What proportion of the samples you collect are from healthy bitches prior to mating compared to those with presumed reproductive disease? Please indicate in percent.

Follow-up questions if options 6.5 b, c are selected. Questions regarding the male dog (6.5.2):

6.5.2.1 How often do you collect swabs from the male reproductive tract? (single choice)

a. Very frequently (more than once per week)

b. Frequently (more than twice per month)

c. Regularly (once per month)

d. Rarely (less than once per month)

6.5.2.2 When do you take swabs from the male reproductive tract? (multiple choice)

a. Suspicion of a bacterial infection

b. Routinely as part of spermatologic examination

c. At owner's/breeder's request

6.5.2.3 Which culture conditions do you request? (single choice)

a. Aerobic only

b. Aerobic and anaerobic

c. Varies depending on the clinical signs

6.7 How many breeders/owners request antimicrobial prescription for their male prior to mating? (single choice)

a. Very many (almost all)

b. Many (more than half)

c. Some (10–50%)

d. Few (sporadic)

e. None

Follow-up question if options 6.7 a, b, c, d are selected.

What are the breeders' rationales for requesting antimicrobial prescription prior to mating? (multiple choice)

a. Clinical signs of reproductive disease

b. Unsuccessful mating in previous cycle

c. Prophylaxis

d. Usual practice

6.5.2.4 Do you use commercial "infertility profiles" (e.g., CHV-PCR, Mycoplasma spp.-PCR, Chlamydia spp.-PCR)? (single choice)

a. Always

b. Regularly

c. In case of unsuccessful previous mating

d. Never

7. How many stud dog owners insist on antimicrobial treatment of the bitch prior to mating? (single choice)

a. Very many (almost all)

b. Many (more than half)

c. Some (10–50%)

d. Few (sporadic)

e. None

f. Don't know

8. Which antimicrobials do you initially administer for suspected reproductive tract infections (e.g., purulent vaginal discharge, pyometra)? (single choice)

a. Beta-lactam antibiotics

b. Fluoroquinolones

c. Cephalosporins

d. Trimethoprim-sulfonamide

9. How long do you typically administer antimicrobials for suspected reproductive tract infections? (single choice)

a. 3–5 days

b. 5–7 days

c. >7 days

10. In which cases do you administer antimicrobials prior to mating? (multiple choice)

a. Generally, if bacterial findings are positive

b. In case of bacterial monoculture

c. In case of high-grade bacterial growth

d. At owner's request

e. Unsuccessful mating with a fertile male in previous cycle

f. Presence of clinical signs in the reproductive tract (e.g., purulent discharge, reddened vaginal mucosa, high neutrophil count in cytology)

g. Always

Follow-up questions if option 10 d is selected. Antimicrobial prescription at the owner's request (10.1 – 10.2):

10.1 What are your reasons for administering antimicrobials to clinically healthy bitches prior to mating at owner's request? (multiple choice)

a. To avoid confrontation/responsibility

b. Unsuccessful mating in previous cycle
c. Uncertainty about bacterial findings
d. Whenever an antimicrobial susceptibility test is available

10.2 Which routes of administration do you use? (single choice)

a. Subcutaneous injection
b. Oral administration
c. Subcutaneous injection followed by oral administration

11. How confident are you in interpreting bacterial culture findings from the bitch's genital flora? (single choice)

a. Very confident
b. Confident
c. Uncertain
d. Very uncertain

12. How frequently do you use antimicrobials in breeding management based on bacteriological results? (single choice)

a. Always (100%)
b. Frequently (>50%)
c. Occasionally (10–50%)
d. Rarely (<10%)
e. Never

13. When do you start antimicrobial treatment if you request antimicrobial susceptibility testing (AST) during routine sampling prior to mating? (multiple choice)

a. At the time of sampling with a non-AST-mandatory antimicrobial (e.g., amoxicillin-clavulanic acid)
b. At the time of sampling with an AST-mandatory antimicrobial (e.g., enrofloxacin)
c. After receiving the susceptibility test results
d. Only if the culture result warrants treatment (e.g., high-grade growth of E. coli in monoculture)

e. Not at all if no clinical signs are present
